# Genome sequencing of *Chlamydia trachomatis* serovars E and F reveals substantial genetic variation

**DOI:** 10.1093/femspd/ftx120

**Published:** 2017-11-24

**Authors:** Thomas Eder, Stefanie Kobus, Sonja Stallmann, Stefanie Stepanow, Karl Köhrer, Johannes H Hegemann, Thomas Rattei

**Affiliations:** 1 Ludwig Boltzmann Institute for Cancer Research, Währinger Straße 13A, 1090 Vienna, Austria; 2 CUBE Division of Computational Systems Biology, Department of Microbiology and Ecosystem Science, University of Vienna, Althanstraße 14, 1090 Vienna, Austria; 3 Institute of Functional Microbial Genomics, Heinrich-Heine-University of Düsseldorf, Universitätsstraße 1, 40225 Düsseldorf, Germany; 4 Biological-Medical Research Center, Heinrich-Heine-University of Düsseldorf, Universitätsstraße 1, 40225 Düsseldorf, Germany

**Keywords:** Chlamydia, genome, adhesins, comparative genomics, evolution

## Abstract

*Chlamydia trachomatis* (Ctr) is a bacterial pathogen that causes ocular, urogenital and lymph system infections in humans. It is highly abundant and among its serovars, E, F and D are most prevalent in sexually transmitted disease. However, the number of publicly available genome sequences of the serovars E and F, and thereby our knowledge about the molecular architecture of these serovars, is low. Here we sequenced the genomes of six E and F clinical isolates and one E lab strain, in order to study the genetic variance in these serovars. As observed before, the genomic variation inside the Ctr genomes is very low and the phylogenetic placement in comparison to publicly available genomes is as expected by *ompA* gene serotyping. However, we observed a large InDel carrying four to five open reading frames in one clinical E sample and in the E lab strain. We have also observed substantial variation on nucleotide and amino acid levels, especially in membrane proteins and secreted proteins. Furthermore, these two groups of proteins are also target for recombination events. One clinical F isolate was genetically heterogeneous and revealed the highest differences on nucleotide level in the *pmpE* gene.

## Teaser

Six *Chlamydia trachomatis* E and F clinical isolates were sequenced and analyzed, which are among the highly abundant and most prevalent serovars in sexually transmitted disease.

## BACKGROUND

The human pathogen *Chlamydia trachomatis* (Ctr) is an obligate intracellular bacterium and the main cause for sexually transmitted diseases worldwide (Bebear and de Barbeyrac 2009) with an increased risk of infertility and ectopic pregnancy when untreated (Paavonen and Eggert-Kruse 1999). It also causes ocular infections up to blindness (Wright, Turner and Taylor 2008). Multiple Ctr strains have been described, which are differentiated based on serotyping of the *ompA* gene (Yuan, Zhang and Watkins 1989). These are linked to various afflictions, such as the ocular strains A–C, the urogenital strains D–K and the strains L1–L3 causing lymphogranuloma venereum. It has been shown that genetic loci are associated with tissue tropism (Fehlner-Gardiner, Roshick and Carlson 2002; Caldwell, Wood and Crane 2003; Carlson, Hughes and Hogan 2004; Carlson, Porcella and McClarty 2005; Gomes, Nunes and Bruno 2006; Jeffrey, Suchland and Quinn 2010; Andersson, Harris and Seth Smith 2016). The serovars E, F and D are the most abundant among the urogenital strains (Bandea, Kubota and Brown 2001). These strains are less virulent than the L serovars (Almeida, Borges and Ferreira 2012), but they are highly prevalent and therefore a substantial factor in human health (FreundM, Buttlar and Giampaolo 1992; Molano, Meijer and Weiderpass 2005; Frej-Madrzak, Teryks-Wołyniec and Jama-Kmiecik 2015). Asymptomatic infections often remain undetected and are therefore not treated. A prominent example for the link between detection and dispersal is the spread of a novel E serovar in 2006 in Sweden (Ripa and Nilsson 2007; Seth-Smith, Harris and Persson 2009; Unemo, Seth-Smith and Cutcliffe 2010). Although the E and F strains are so abundant, they are not so easy to handle in lab culture like for example the virulent L-strains and consequently our knowledge of their genomic capabilities is still limited. The whole-genome analysis by Harris, Clarke and Seth-Smith (2012) covers only seven E and four F genomes. In order to better understand the pathogenicity of these strains and their natural variability, genome sequences of further representative clinical isolates are highly important. We have sequenced and comparatively analyzed the genomes of seven, mainly clinical, Ctr E and F samples from Germany.

## GENOME RECONSTRUCTION

**Figure 1.**
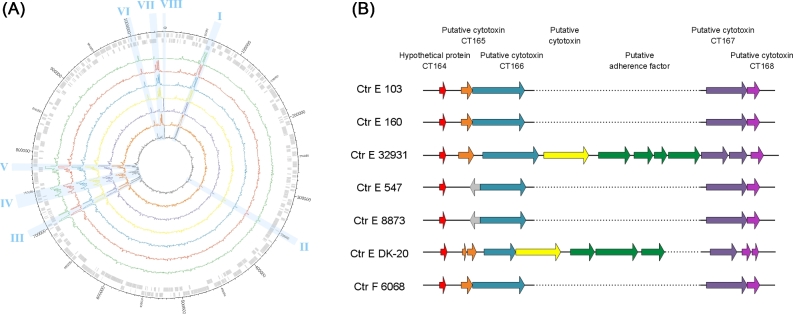
SNP density and the large insert. (**A**) This plot shows the SNP densities over the genome sequences of all samples. On the two outermost circles, the genes are plotted in gray, first the forward then the reverse ones. Starting from the outside the genomes are E 103 green, E 160 red, E 32931 blue, E 547 yellow, E 8873 purple, E DK-20 orange and F 6068 gray. The light-blue areas indicate regions with a high SNP density affecting the following proteins. I: Three hypothetical proteins CT_049, CT_050 and CT_051; II: The hypothetical protein CT_310 and the V-type ATP synthase subunit E CT_311; III: Hypothetical protein CT_619 and hypothetical protein CT_622; IV: The intergenic region between formyltetrahydrofolate synthetase CT_649, recombinase RecA CT_650 and a hypothetical protein CT_651; V: major outer membrane protein CT_681; VI: membrane protein CT_852; VII: outer membrane protein PmpE and PmpF, CT_869 and CT_870; VIII: outer membrane protein PmpH CT_872 and hypothetical protein CT_873. (**B**) Scheme of the region surrounding the large insert plus the predicted annotation of the genes and their homologs in *C. trachomatis* D/UW-3/CX (CT numbers). Whereas CT_164 is present in all seven samples, the ORF representing CT_165 shows changes in E DK-20 and E 32 931, as well as in E 547 and E 8873, where it is replaced by an ORF on the other strand. CT_166 is also affected by the insert in E 32931 and DK-20. The first ORF of the insert is predicted to be a cytotoxin, followed by three or four putative adherence factors. The insert in E DK-20 is around 900 bp shorter and in E DK-20 and E 32931 either CT_167 or CT_168 is disturbed.

We sequenced 8875 105 paired-end sequence reads (Table 1; Table S1; Supplementary methods) from six Ctr clinical samples and one Ctr lab strain (E DK-20). These were assembled into one closed chromosome and one plasmid for each sample. We compared the closed genomes to one E (E 150), one F (F SW4) and one D (UW-3/CX) reference sequence which are publicly available (Fig. S1; Table S2, Supporting Information). Eight regions have particularly high SNP densities (Fig. 1A; Table S3, Supporting Information). Only six SNPs and one deletion were found in only four of the plasmid sequences (Ctr E 32931, 8873, DK-20 and F 6068) (Table S4, Supporting Information). The deletion and two SNPs have been observed in smaller and larger fractions of the sequenced plasmids (Table S4), so the distribution is not always homogeneous. From the seven samples investigated in this study, six showed evidence for single infection but Ctr F 6068 consisted of a heterogeneous population. Its reconstructed genome represents the major component but for 70 positions we see SNPs with around 21% coverage, meaning that this sample had a subpopulation of this percentage (Table S5, Supporting Information).

**Table 1.** Overview on samples and genomes.

Data set	Strain name/ isolate	Serotype	Country	City	Source	Year of isolation	Clinical manifestation	Nr passages	Length of chromosome in bp	Length of plasmid in bp	CDS	Accession
CtrE-103	103	E	GER		Female	1992		14	1043 019	7502	970	CP015294, CP015295
CtrE-160	160	E	GER		Female	1995		11	1043 007	7502	968	CP015296, CP015297
CtrE-32 921	32921	E	GER	Stadtroda	Vagina, 20-year-old female	2003		4	1048 917	7502	970	CP015302, CP015303
CtrE-547	547	E	GER		Female	1991		12	1043 003	7502	963	CP015298, CP015299
CtrE-8873	8873	E	GER	Jena	Female	1998	Urethritis	9	1042 717	7493	970	CP015300, CP015301
CtrE-DK-20	DK-20	E	GB	London	Institute of Ophthalmology	1977		Unknown	1048 033	7502	967	CP015304, CP015305
					Reference strain (Treharne, Darougar and Jones 1977)							
CtrF-6068	6068	F	GER	Jena	Urethra, 19-year-old female	1997	Urethritis	10	1042 738	7493	969	CP015306, CP015307

Overview about *C. trachomatis* sample names, sample metadata, sequencing characteristics and genome summary.

## CHROMOSOMAL INSERTION/DELETION (InDel)

We compared the reconstructed genomes not only in between each other but also to the publicly available Ctr genomes (Table S2). We identified a large InDel (around 5 kb long) in Ctr E DK-20 and E 32931. In Ctr E 32931, the region is 5913 nucleotides long and consists of five open reading frames (ORFs), whereas the region in Ctr E DK-20 is, with 5041 nucleotides and four ORFs, slightly shorter (Fig. 1B). The InDel is in both cases located between the putative cytotoxins CT_166 and CT_167. We observed variation in the predicted gene structure in close proximity to the InDel. There is a frameshift predicted in E 32931 in CT_167 and also in E DK-20 in CT_165 and CT_168. We found the complete E 32931 InDel with 100% identity only in Ctr E SotonE8. With a high similarity for the E 32931 InDel and 100% identity for the E DK-20 InDel, we find it also in the *in vitro*-generated strains Ctr RC-L2(s)/3, RC-J(s)/122, RC-F/69, RC-F(s)/342 and RC-F(s)/852 (Jeffrey, Suchland and Eriksen 2013). With only a few mismatches the InDels were also found in Ctr serotypes H and J, which are also infecting the urogenital tract (Carlson, Hughes and Hogan 2004), annotated as cytotoxin genes in UW-36 and UW-4. The 3΄ part of around 1720 nucleotides is also present in A, B and C serotypes (A2497, A/363, A/HAR-13, A/7249, A/5291, B/Jali20/OT, B/TZ1A828/OT and C/TW-3) and it is also described as cytotoxin genes in AP2, Har-13 and TW-3 (Carlson, Hughes and Hogan 2004). Regions homologous to the InDel were also found in *Chlamydia suis* and *C. muridarum* with an average amino acid sequence identity of 70%.

## PHYLOGENETIC RECONSTRUCTION

The maximum-likelihood phylogenetic tree of the *ompA* gene shows a clear separation of the E and F serovars and the D reference (Fig. S2, Supporting Information). As expected, the reconstructed samples are closely located to their respective reference genomes. We also used the complete genome sequences of our samples together with 69 public available genomes including the 50 genomes investigated by Harris, Clarke and Seth-Smith (2012) for phylogenetic reconstruction (Fig. S3, Supporting Information). All E and F serovars are in their respective subtrees, which is consistent with the *ompA* phylogeny and therefore we can exclude recombination in *ompA* for the seven reconstructed genomes. Interestingly, the only E serovar, in which we found the large InDel (Ctr E SotonE8), is the nearest neighbor to Ctr E 32931. This could be an indication that their common ancestor might also have carried this InDel if it was not acquired by recombination.

## RECOMBINATION

Based on the SNPs, we observed significant evidence for recombination within the seven reconstructed genomes and the three reference genomes (E-150, F-SW4 and D/UW-3/CX). The EqualAngle tree reconstruction shows connections in between the serovar E cluster and E 8873 as well as the F serovars (Fig. S4, Supporting Information). Recombination events were found inside the newly sequenced E and F serovars (Fig. S5; Table S6, Supporting Information).

## EVOLUTIONARY SELECTION

We investigated the genes with high Ka/Ks ratios in between the seven genomes and the three reference genomes, which are thought to be those who evolve under positive selection. After ranking and statistical testing (Tables S7 and S8, Supporting Information), the top-ranking ratios were CT_868, CT_867 (Misaghi, Balsara and Catic 2006) and CT_694 (Bullock, Hower and Fields 2012), which are all experimentally verified type III secreted, effector proteins. CT_089 (Fields and Hackstadt 2000) and CT_116 (Subtil, Delevoye and Balañá 2005) are predicted to be secreted by the type III secretion system of Ctr. CT_198 with its function for transmembrane transport is involved in transport or possibly presented outside *Chlamydia*, so the high Ka/Ks ratio is very plausible. The endonuclease CT_157 has also been seen as highly polymorphic in Kari, Whitmire and Carlson (2008), and the authors suspect that it is involved in pathogenicity of Ctr strains. The hypothetical protein CT_105 is likely to be a type III substrate of Ctr (da Cunha, Milho and Almeida 2014). In contrast, the high ratios in the hypothetical proteins CT_168 and CT_244 point toward possible candidates for being secreted effectors, membrane proteins or being involved in transport to the endosome or host cell. The Ka/Ks ratio in the *pmp* gene family is below 1 for *pmpD* and *pmpI*, and also for *pmpA* and *pmpB* and around 1 for *pmpC* (Table S9, Supporting Information).

## DISCUSSION

We sequenced and reconstructed the complete genomes of seven Ctr E and F strains, six of them from clinical samples and one from a lab strain (E DK-20). We observed highest diversity at loci coding for hypothetical proteins, as well as *ompA* and the *pmpE* and *pmpF* genes. These loci are in agreement with increased SNP and homoplasy density regions found within other Ctr serovars (Harris, Clarke and Seth-Smith 2012). Besides hypothetical proteins, mainly membrane proteins and secreted proteins show high numbers of SNPs. This strengthens the assumption of higher evolutionary variability of genes involved in interactions with the host. The phylogenetic placement agrees overall with the tree previously presented (Harris, Clarke and Seth-Smith 2012). Compared to the phylogenetic tree of the *ompA* gene, the whole-genome-based tree indicates that especially the lab strain Ctr E DK-20 has a higher genetic distance to the other E serovars. Ctr E 8873 has diverged most early from the other E serovars. We found evidence for several recombination events, covering genes with diverse functions including several membrane-related ones. Similar to all other E and F strains, the subpopulation in F 6068 differs most in the *pmpE* gene which indicates that the genomic variation is focused on this particular membrane protein. Co-infections of Ctr serovars (Bandea, Kubota and Brown 2001; Jurstrand, Falk and Fredlund 2001; Lee, Park and Kim 2006; Gharsallah, Frikha-Gargouri and Sellami 2012; Zhang, Zhao and Wang 2012; Rodriguez-Dominguez, Gonzalez-Alba and Puerta 2015; Gallo Vaulet, Entrocassi and Portu 2016) are the prerequisite for recombination. In the F 6068 sample, we could detect a co-infection. However, the genetic difference between the two genotypes was small. A large genomic InDel of about 5 kb in two samples seems to originate from the last common ancestor of *Chlamydia suis*, *C. muridarum* and Ctr. It might have been acquired via recombination (Jeffrey, Suchland and Eriksen 2013). The full-length InDel is only present in two samples from this study (E DK-20 and E 32931) and in Ctr E SotonE8, in the H and J serovars and in five *in vitro* artificially generated strains (Jeffrey, Suchland and Eriksen 2013). Small parts of the InDel have also been previously described (Carlson, Hughes and Hogan 2004; Unemo, Seth-Smith and Cutcliffe 2010). It encodes a large cytotoxin gene in *C. muridarum*, whereas in the reconstructed E serovars it is split into four or five ORFs, predicted to be cytotoxin genes and one adherence factor. High Ka/Ks ratios indicate that selection favors changes in the amino acid sequences. As expected, the genes with the highest Ka/Ks ratios are mainly type III secreted proteins (known and predicted), hypothetical or involved in the transport to the endosome or host. Statistical evidence strengthens the assumption of a more variable secretome, compared to the majority of proteins. In summary, our study demonstrates a substantial genomic variation in the abundant Ctr E and F strains. These loci and genes may have high impact on the pathogenicity of Ctr, and will be relevant for the development of novel diagnostic tools and vaccines.

## SUPPLEMENTARY DATA

Supplementary data are available at *FEMSPD* online.

***Conflict of Interest*.** None declared.

Supplementary material Supplementary data are available at *FEMSPD* online.
