# Supplementary material for: Genome sequencing of Chlamydia trachomatis serovars E and F reveals substantial genetic variation
Source: Pathog Dis. 2017 Nov 24;75(9):ftx120. doi: 10.1093/femspd/ftx120 (PMC5827700; doi:10.1093/femspd/ftx120)
Supplement: Supplementary material — Supplementary data are available at FEMSPD online. [file ftx120_supp.zip › pad_suppl_methods_plain.docx]

**Supplementary methods**

**Clinical *Chlamydia trachomatis* isolates**

The six clinical *C. trachomatis* isolates 32921, 6068, 160, 103, 8873, 547 and the serovar E prototype strain DK-20 (Table 1) were obtained from the sample archive at the Institut für Medizinische Mikrobiologie at the Jena University Hospital in Jena (Germany). The clinical samples were originally obtained from clinical specimens, isolated and amplified via several passages and stored at −80°C after original isolation. For revitalization, the samples were propagated in cycloheximide-treated epithelial African green monkey (BGM) cells (without antibiotics) in shell vials and then inoculated on to 25 cm2 tissue culture flasks. The final amplification passage was done with HEp-2 cells.

For DNA isolation, bacterial cells were pelleted and incubated with DNase and subsequently with RNase A to remove host cell DNA and RNA respectively. Cells were again pelleted, re-suspended in a Lysozyme and Proteinase K solution and incubated over night at 37°C, and with fresh Proteinase K for an additional 2 hours at 55°C. After phenol/chloroform/isoamylalcohol extraction the DNA was pelleted by addition of isopropanol, followed by an ethanol wash of the DNA pellet. DNA was solved in water and its concentration determined by NanoDrop. Each *C. trachomatis* DNA sample was analyzed by real time PCR, targeting the *C. trachomatis* genomic *ct875* (MOMP) and *ct694* loci, the human GAPDH and Actin coding genes, as well as the 16S *Mycoplasma* locus.

**DNA extraction and sample information**

Approximately 1 ng/µL DNA concentration and fragment lengths around 1kB were adjusted for library preparation (Table S10).

**Library preparation and NGS sequencing**

We prepared NGS libraries from six *C. trachomatis* clinical samples with the Nextera DNA Library Preparation Kit (Illumina, FC-121-1030) together with the Nextera Index Kit (Illumina, FC-121-1011) following the manufacturer’s protocols (#15027987, Rev.B) with 50 ng genomic DNA as input and with an additional bead purification step of the final library with a bead to template ratio of 0.6x. The library preparation of the *C. trachomatis* lab strain (E DK-20) was modified because of a small final library fragment size of 241 bp. We adapted the input amount from 50 ng to 70 ng genomic DNA and the incubation time of the tagmentation step in the Nextera protocol from 5 min to 1 min and added an additional bead purification step of the final library with a bead to template ratio of 0.7x. These modifications led to an average library size of 491 bp. The NGS libraries of the clinical samples are concentrated from 0.75 ng/µL to 1.18 ng/µL and showed a mean fragment length distribution between 831 bp and 1170 bp. The libraries were quantified using the Qubit dsDNA HS assay (ThermoFisher) and the fragment size distribution was determined with 2100 Bioanalyzer instrument using a HighSensitivity DNA Chip (Agilent). For sequencing we pooled the seven libraries equimolarly. NGS sequencing of the isolates was performed on an Illumina MiSeq instrument using the 600-cycle format of the MiSeq Reagent Kit v3 (Illumina, #MS-102-3003) generating (2x300 bp) paired-end reads.

**Bioinformatic genome reconstruction**

The initial quality check of the raw sequence reads was done via the FastQC (http://www.bioinformatics.babraham.ac.uk/projects/fastqc; version 0.11.1) tool. Based on the observed length and quality patterns, the preprocessing was done with PRINSEQ-lite [1] (version 0.20.4). The following parameters were applied: for quality trimming left and right 20, minimal quality mean 20, minimum length 50 and the maximum number of N’s was set to 20 percent. To remove host cell derived sequence reads, the preprocessed reads were mapped via BWA [2] (version 0.7.10) against the Human-reference sequence (Genome Reference Consortium Human Build 38 patch release 2 (GRCh38.p2) Accession: GCF_000001405.28). The remaining, non-mapped high quality sequence reads were used for *de-novo* assembly with SPAdes [3] (version 3.1.0) and MaSuRCA [4] (version 2.3.0rc1) in parallel. The following parameters were used in SPAdes: -k 63,93,113,127 and in MaSuRCA: GRAPH_KMER_SIZE auto, USE_LINKING_MATES 1, CA_PARAMETERS ovlMerSize 30, cgwErrorRate 0.25, ovlMemory 8GB, KMER_COUNT_THRESHOLD 1 and DO_HOMOPOLYMER_TRIM 0. In addition to the *de-novo* assembly the non-mapped high quality sequence reads were used in a comparative assembly run with AMOScmp [5] (version 3.1.0) with default parameters and the reference sequence *C. trachomatis* E-150 (Accession: NC_017439) for the E serovars and *C. trachomatis* F-SW4 (Accession: NC_017951) for the F serovar. The contigs from the two *de-novo* and the comparative assembly runs were aligned against the two reference sequences via Mauve [6] (version 2.3.1). Manual contig joining was done based on this multiple sequence alignment. In order to join the two contigs in *C. trachomatis* E 32931 and the three contigs in *C. trachomatis* E DK-20 produced by AMOScmp, we used the sequences of the *de-novo* scaffolds to bridge the gaps (Fig. S6; Table S11).

To check the manually modified regions the closed chromosome sequences were used as references for a mapping run of the preprocessed sequence reads with BWA. *Mycoplasma* reference sequences were included in this step, in order to remove contaminant *Mycoplasma* sequence reads. The following three species were used in the mapping step: *Mycoplasma hominis* (Accession: NC_013511), *Mycoplasma fermentans* (Accession: NC_014552) and *Mycoplasma hyorhinis* (Accession: NC_022807). After manual refinement, the final chromosome sequences were again aligned against the two reference sequences plus *C. trachomatis* D/UW-3/CX (Accession: NC_000117.1) via Mauve. This tool was also used for extracting SNPs and short indels. In an additional step the final multiple alignment was checked manually for misassembled indel positions by going over each position via SeaView [7] (version 4.4.1) and comparing it to the mapped reads visualized via Tablet [8] (version 1.13.07.31). The simplified bioinformatic work-flow is shown in Figure S7.

The seven closed *C. trachomatis* chromosomes were annotated with the RAST-Server [9] with default settings and genetic code 11 for bacteria. This tool was also used to calculate the bi-directional best hits of the ORFs in the seven genomes. To test the reconstructed chromosomes for completeness we used three tools, namely tRNAscan [10] (version 1.23) to predict the number of tRNAs, REAPR [11] (version 1.0.18) to check for assembly errors and CheckM (version 1.0.5) [12].

**Comparative genomics**

The multiple alignment of the seven reconstructed genomes together with the two reference genomes *C. trachomatis* E-150 and F-SW4 used for the comparative assembly and *C. trachomatis* D/UW-3/CX as out-group, was used to extract the SNPs. The maximum likelihood phylogeny, based on these SNPs and based on the *ompA* gene was calculated with Raxml [13] (version 8.2.0). For the SNPs the ASC_GTRGAMMA model with lewis correction and for *ompA* the GTRGAMMA model was used. All phylogenetic trees were calculated with 1,000 bootstrap replicates each. In addition we used 69 (Table S12) publicly available *C. trachomatis* genomes, including the 50 genomes investigated in Harris et al. [14], to reconstruct the phylogenetic relationship between those genomes and our samples. We used Mauve and Raxml, as previously described, with the ASC_GTRGAMMA model with lewis correction and 1,000 bootstrap replicates. To investigate the *C. trachomatis* plasmids, the high quality sequence reads were mapped via BWA against the *C. trachomatis* E Bour plasmid sequence (Accession: NC_020947). Indels and SNPs were called via the GATK [15] (version 3.3) UnifiedGenotyper. The Ka/Ks calculations for all annotated genes, were done via the KaKs_Calculator_2.0 [16] (version 1.2) with -c 11 and -m MYN, parameters. The input was based on the Mauve multiple alignment from where the homologs of all genes were extracted via ParaAT [17] with the parameter -c 11 for the bacterial genetic code. In order to test if we see a statistical significant relation between secretion and the mutation rate of a protein we selected secreted proteins or ones which are predicted to be secreted and all other proteins. We then also separated all proteins into proteins with two or more non-synonymous SNPs and proteins with less or none non-synonymous SNPs. We applied the chi-square-test and it was significant (p-value = 0.006). The final chromosomes were tested for recombination via splitstree [18] (version 4.11.3) and the recombination sites where identified with ClonalFrameML [19] (version 1.-178). For ClonalFrameML we utilized the already calculated phylogenetic tree based on all SNPs from the Mauve alignment. The sequence alignments for this tool were calculated via MAFFT [20] (version 7.245) from the complete genome sequences. The specific blast [21] searches for occurrence of the large inserts in other *C. trachomatis* serovars or *Chlamydia* species was executed against the NCBI nr and nt databases. To check the high quality sequenced reads for any contamination outside Mycoplasma, we used DeconSeq [22] (version 0.4.3). As databases we provided the seven *C. trachomatis* genomes plus the *C. trachomatis* E Bour plasmid sequence (Accession: NC_020947), the three *Mycoplasma* species: *Mycoplasma hominis* (Accession: NC_013511), *Mycoplasma fermentans* (Accession: NC_014552) and *Mycoplasma hyorhinis* (Accession: NC_022807) and the human reference sequence (Genome Reference Consortium Human Build 38 patch release 2 (GRCh38.p2) Accession: GCF_000001405.28). With the reads from these organisms removed, we started a RAPSearch [23, 24] run (version 2.23) against the NCBI nr database. The results were loaded into MEGAN [25] (version 5.10.5) and the resulting taxonomic compositions were then compared within this tool.

**Availability of data and materials**

The datasets supporting the conclusions of this article are available on GenBank under the Bioproject: PRJNA316787. The accessions to the seven chromosomes and the corresponding plasmids are: CP015294 & CP015295 for E 103, CP015296 & CP015297 for E 160, CP015298 & CP015299 for E 547, CP015300 & CP015301 for E 8873, CP015302 & CP015303 for E 32931, CP015304 & CP015305 for E DK-20 and CP015306 & CP015307 for F 6068.

**References**

1. Schmieder, R. and R. Edwards, *Quality control and preprocessing of metagenomic datasets.* Bioinformatics, 2011. **27**(6): p. 863-4.

2. Li, H. and R. Durbin, *Fast and accurate short read alignment with Burrows-Wheeler transform.* Bioinformatics, 2009. **25**(14): p. 1754-60.

3. Bankevich, A., et al., *SPAdes: a new genome assembly algorithm and its applications to single-cell sequencing.* J Comput Biol, 2012. **19**(5): p. 455-77.

4. Zimin, A.V., et al., *The MaSuRCA genome assembler.* Bioinformatics, 2013. **29**(21): p. 2669-77.

5. Pop, M., et al., *Comparative genome assembly.* Brief Bioinform, 2004. **5**(3): p. 237-48.

6. Darling, A.E., B. Mau, and N.T. Perna, *progressiveMauve: multiple genome alignment with gene gain, loss and rearrangement.* PLoS One, 2010. **5**(6): p. e11147.

7. Gouy, M., S. Guindon, and O. Gascuel, *SeaView version 4: A multiplatform graphical user interface for sequence alignment and phylogenetic tree building.* Mol Biol Evol, 2010. **27**(2): p. 221-4.

8. Milne, I., et al., *Using Tablet for visual exploration of second-generation sequencing data.* Brief Bioinform, 2013. **14**(2): p. 193-202.

9. Aziz, R.K., et al., *The RAST Server: rapid annotations using subsystems technology.* BMC Genomics, 2008. **9**: p. 75.

10. Lowe, T.M. and S.R. Eddy, *tRNAscan-SE: a program for improved detection of transfer RNA genes in genomic sequence.* Nucleic Acids Res, 1997. **25**(5): p. 955-64.

11. Hunt, M., et al., *REAPR: a universal tool for genome assembly evaluation.* Genome Biol, 2013. **14**(5): p. R47.

12. Parks, D.H., et al., *CheckM: assessing the quality of microbial genomes recovered from isolates, single cells, and metagenomes.* Genome Res, 2015. **25**(7): p. 1043-55.

13. Stamatakis, A., *RAxML-VI-HPC: maximum likelihood-based phylogenetic analyses with thousands of taxa and mixed models.* Bioinformatics, 2006. **22**(21): p. 2688-90.

14. Harris, S.R., et al., *Whole-genome analysis of diverse Chlamydia trachomatis strains identifies phylogenetic relationships masked by current clinical typing.* Nat Genet, 2012. **44**(4): p. 413-9, S1.

15. McKenna, A., et al., *The Genome Analysis Toolkit: a MapReduce framework for analyzing next-generation DNA sequencing data.* Genome Res, 2010. **20**(9): p. 1297-303.

16. Zhang, Z., et al., *KaKs_Calculator: calculating Ka and Ks through model selection and model averaging.* Genomics Proteomics Bioinformatics, 2006. **4**(4): p. 259-63.

17. Zhang, Z., et al., *ParaAT: a parallel tool for constructing multiple protein-coding DNA alignments.* Biochem Biophys Res Commun, 2012. **419**(4): p. 779-81.

18. Huson, D.H. and D. Bryant, *Application of phylogenetic networks in evolutionary studies.* Mol Biol Evol, 2006. **23**(2): p. 254-67.

19. Didelot, X. and D.J. Wilson, *ClonalFrameML: efficient inference of recombination in whole bacterial genomes.* PLoS Comput Biol, 2015. **11**(2): p. e1004041.

20. Katoh, K. and D.M. Standley, *MAFFT multiple sequence alignment software version 7: improvements in performance and usability.* Mol Biol Evol, 2013. **30**(4): p. 772-80.

21. Altschul, S.F., et al., *Basic local alignment search tool.* J Mol Biol, 1990. **215**(3): p. 403-10.

22. Schmieder, R. and R. Edwards, *Fast identification and removal of sequence contamination from genomic and metagenomic datasets.* PLoS One, 2011. **6**(3): p. e17288.

23. Ye, Y., J.H. Choi, and H. Tang, *RAPSearch: a fast protein similarity search tool for short reads.* BMC Bioinformatics, 2011. **12**: p. 159.

24. Zhao, Y., H. Tang, and Y. Ye, *RAPSearch2: a fast and memory-efficient protein similarity search tool for next-generation sequencing data.* Bioinformatics, 2012. **28**(1): p. 125-6.

25. Huson, D.H., et al., *Integrative analysis of environmental sequences using MEGAN4.* Genome Res, 2011. **21**(9): p. 1552-60.
