# Supplementary material for: Genome sequencing of Chlamydia trachomatis serovars E and F reveals substantial genetic variation
Source: Pathog Dis. 2017 Nov 24;75(9):ftx120. doi: 10.1093/femspd/ftx120 (PMC5827700; doi:10.1093/femspd/ftx120)
Supplement: Supplementary material — Supplementary data are available at FEMSPD online. [file ftx120_supp.zip › pad_suppl_tables_plain.docx]

**Table S1:** Sequencing characteristics, genome stats and accessions

| Genome | Read pairs | Quality filter passed | Estimated coverage | Mapped to human | Length of chromosome in bp | Length of plasmid in bp | CDS | rRNA | tRNA | Accession |
| --- | --- | --- | --- | --- | --- | --- | --- | --- | --- | --- |
| CtrE-103 | 1,219,272 | 97.73% | 625x | 5.30% | 1,043,019 | 7,502 | 970 | 2x(16S 23S 5S) | 37 | CP015294, CP015295 |
| CtrE-160 | 1,489,152 | 98.79% | 680x | 11.54% | 1,043,007 | 7,502 | 968 | 2x(16S 23S 5S) | 37 | CP015296, CP015297 |
| CtrE-32921 | 1,053,824 | 98.21% | 558x | 3.43% | 1,048,917 | 7,502 | 970 | 2x(16S 23S 5S) | 37 | CP015302, CP015303 |
| CtrE-547 | 1,020,202 | 97.51% | 454x | 12.69% | 1,043,003 | 7,502 | 963 | 2x(16S 23S 5S) | 37 | CP015298, CP015299 |
| CtrE-8873 | 993,224 | 96.91% | 472x | 9.80% | 1,042,717 | 7,493 | 970 | 2x(16S 23S 5S) | 37 | CP015300, CP015301 |
| CtrE-DK-20 | 1,611,495 | 99.46% | 739x | 18.81% | 1,048,033 | 7,502 | 967 | 2x(16S 23S 5S) | 37 | CP015304, CP015305 |
| CtrF-6068 | 1,487,936 | 98.40% | 799x | 2.96% | 1,042,738 | 7,493 | 969 | 2x(16S 23S 5S) | 37 | CP015306, CP015307 |

**Table S2**: List of all strain names, reference genomes, host genomes, plasmids and genes and accessions. The genome sequences of the bacterial species were used for contamination removal and the human genome sequence to remove host derived sequence reads. The genomes of the *C. trachomatis* strains as well as the *C. trachomatis* plasmid and genes were used for genome comparison on whole genome and gene level.

| **Bacterial species** | **Accession** |
| --- | --- |
| *Mycoplasma hominis* | NC 013511 |
| *Mycoplasma fermentans* | NC 014552 |
| *Mycoplasma hyorhinis* | NC 022807 |
| **Human genome** | **Accession** |
| Genome Reference Consortium Human Build 38 patch release 2 (GRCh38.p2) | GCF 000001405.28 |
| ***C. trachomatis* strains** | **Accession** |
| E-150 | NC 017439 |
| F-SW4 | NC 017951 |
| D/UW-3/CX | NC 000117 |
| E SotonE8 | NC_020942 |
| RC-L2(s)/3 | NC_021897 |
| RC-J(s)/122 | NC_021891 |
| RC-F/69 | NC_021895 |
| RC-F(s)/342 | NC_021890 |
| RC-F(s)/852 | NC_021888 |
| A2497 | NC_016798 |
| A/363 | NC_020966 |
| A/HAR-13 | NC_007429 |
| A/7249 | NC_020944 |
| A/5291 | NC_020939 |
| B/Jali20/OT | NC_012686 |
| B/TZ1A828/OT | NC_012687 |
| C/TW-3 | NC_023060 |
| ***C. trachomatis* plasmid** | **Accession** |
| E Bour plasmid | NC 020947 |
| ***C. trachomatis* genes** | **Accession** |
| UW-36 | AY648001 |
| UW-4 | AY647999 |
| P2 | AY647993 |
| Har-13 | AY647992 |
| TW-3 | AY647994 |

**Table S3: Polymorphic regions from Figure 1A.** Regions with highest SNP densities as indicated in Figure 1A.

| **Region with high SNP density (>45/kB)** | **Genes** | **Description** |
| --- | --- | --- |
| I | CT_049 | Pls-1 [1], Pmp-like secreted, putative effector protein [2] |
|  | CT_050 | Pls-2 [1], Pmp-like secreted, putative effector protein [2] |
|  | CT_051 | Pls-3, Pmp-like secreted [1] |
| II | CT_310 | V-type ATP synthase subunit E |
|  | CT_311 | hypothetical protein, has been shown to move into the host nucleus and accumulates there [3] |
| III | CT_619 | hypothetical protein, interaction with host ESCRT machinery [4] |
|  | CT_622 | hypothetical protein |
| IV | Intergenic 3’ of CT_649 | formyltetrahydrofolate synthetase |
|  | CT_650 | recombinase RecA |
|  | intergenic 5’ of CT_651 | putative exported lipoprotein |
| V | CT_681 | major outer membrane protein (MOMP) |
| VI | CT_852 | hypothetical protein; recognized by more than 50 % of patient sera tested [5-7] |
| **Low SNP density (<=45/kB) in E DK-20 and F 6068:** | | |
| VII | CT_869 | outer membrane protein PmpE |
|  | CT_870 | outer membrane protein PmpF |
| **High SNP density (>45/kB) in E DK-20 and F 6068:** | | |
| VIII | CT_872 | outer membrane protein PmpH |
|  | Intergenic region | between CT_872 and CT_873 |
|  | CT_873 | hypothetical protein |

**Table S4: Plasmid differences.** This table shows the position in the plasmid reference sequence, the reference base or sequence and the alternative found in the mapping. For the four samples with SNPs or the deletion, the percentage of reads showing this variation is indicated. Then the position is linked to an annotated gene or a gene in closer proximity plus its function/product.

| **Position** | **Reference** | **Alternative** | **E-32931** | **E-8873** | **E-DK-20** | **F-6068** | **SNP/DEL in gene** | **Product** |
| --- | --- | --- | --- | --- | --- | --- | --- | --- |
| 120 | C | A |  |  | 100% |  | 5‘ BOUR p1 | putative virulence plasmid integrase |
| 1147 | G | T |  | 100% | 100% | 94.55% | BOUR p2 | putative virulence plasmid integrase |
| 1286 | C | T |  | 100% |  |  | BOUR p2 | putative virulence plasmid integrase |
| 2523 | C | T |  | 100% |  |  | BOUR p3 | putative helicase 2C DnaB family |
| 4479 | C | A | 100% |  |  |  | BOUR p4 | conserved hypothetical  virulence plasmid protein |
| 4607 | GGAACAATTA | G |  | 100% |  | 88.80% | BOUR p4 | conserved hypothetical  virulence plasmid protein |
| 7463 | T | A |  | 10.40% |  |  | 3‘ BOUR p8 | conserved hypothetical  virulence plasmid protein |

**Table S5: Sub-population in CtrF-6068.** The number of SNPs in CtrF-6068 in a sub-population of 21,26%

| **Gene** | **Number of SNPs** |
| --- | --- |
| *gatB* | 1 |
| *phoH* | 2 |
| *dapA* | 3 |
| *lysC* | 1 |
| *asd* | 3 |
| *dapB* | 1 |
| *aroA* | 1 |
| *aroL* | 1 |
| *aroB* | 2 |
| CT_375 | 2 |
| pgi | 4 |
| *aroG* | 1 |
| CT_383 | 1 |
| CT_384 | 2 |
| CT_387 | 2 |
| CT_391 | 2 |
| *hrcA* | 2 |
| CT_456 | 3 |
| CT_663 | 1 |
| *gpdA* | 1 |
| CT_861 | 1 |
| CT_863 | 1 |
| CT_868 | 2 |
| *pmpE* | 11 |
| *pmpF* | 2 |
| *pmpG* | 2 |
| *pmpH* | 2 |
| CT_873 | 3 |
| CT_875 | 1 |

**Table S6: Recombination.** Recombination events could be identified in these genomic regions or genes in the respective strains.

| ***C. trachomatis* strain** | **Genomic region / gene** | **Description** |
| --- | --- | --- |
| E 32931 | intergenic 3’ CT_146 | DNA ligase |
|  | intergenic 5’ and in CT_147 | putative integral membrane protein, CT147 can distinguish women with acute *C. trachomatis* infection from those with tubal factor infertility and normal fertility [8] |
|  | CT_161 | heterologously secreted, likely a T3SS effector protein [9] |
|  | CT_162 | uncharacterized protein |
| E 103 | CT_675 | protein-arginine kinase |
|  | CT_745 | protoporphyrinogen oxidase |
| DK-20 | CT_048 | SAM-dependent methyltransferase |
|  | CT_049 | cell wall associated hydrolases domain-containing protein |
|  | CT_051 | heterologously secreted, likely a T3SS effector protein [9] |
|  | CT_096 | translation initiation factor IF-2 |
|  | CT_105 | uncharacterized protein |
|  | CT_159 | hypothetical protein |
|  | CT_160 | hypothetical protein |
|  | CT_166 | adherence factor |
|  | four ORFs in the insertion | putative cytotoxin, putative adherence factors |
|  | CT_171 | tryptophan synthase alpha chain |
|  | intergenic 3’ CT_213 | ribose-5-phosphate isomerase |
|  | intergenic 5’ CT_214 | candidate inclusion membrane protein |
|  | CT_860 | conserved hypothetical serine-rich protein |
| F 6068 | intergenic 3’ CT_141 | protein translocase |
|  | intergenic 5’ CT_142 | heterologously secreted, likely a T3SS effector protein [9] |
|  | intergenic 3’ CT_144 | heterologously secreted, likely a T3SS effector protein [9] |
|  | intergenic 5’ CT_147 | putative integral membrane protein |
|  | CT_633 | delta-aminolevulinic acid dehydratase |

**Table S7: Ka/Ks ratios.** The genes with high Ka/Ks ratios (min. 2), with their LocusTag, gene product and description. The first three are experimentally verified secreted effector proteins and CT_098, CT_116 and CT_105 are predicted to be secreted.

| **Ka/Ks ratio** | **LocusTag** | **Gene product** | **Description** |
| --- | --- | --- | --- |
| 4.32036 | CT_867 | Deubiquitinase and deneddylase Dub2 | Effector |
| 4.20918 | CT_694 | Hypothetical protein | Effector |
| 4.10522 | CT_868 | Deubiquitinase and deneddylase Dub1 | Effector |
| 3.21919 | CT_244 | Hypothetical protein |  |
| 2.6095 | CT_168 | Hypothetical protein |  |
| 2.54279 | CT_089 | Low calcium response E | Predicted: protein secretion by the type III secretion system |
| 2.26665 | CT_157 | Phospholipase D endonuclease superfamily | Endonuclease activity |
| 2.12628 | CT_116 | Inclusion membrane protein E | Secreted, probably via type III secretion system |
| 2.03917 | CT_105 | Hypothetical protein | Predicted: protein secretion by the type III secretion system |
| 2.00175 | CT_198 | Oligopeptide binding protein | Transmembrane transport |

**Table S8:** Effector proteins used for chi-square test.

| **LocusTag** | **Predicted function** |
| --- | --- |
| CT_005 | Inc protein |
| CT_036 | Inc protein |
| CT_049 | Pls1 |
| CT_050 | Pls2 |
| CT_053 |  |
| CT_058 | Inc protein |
| CT_083 | Inc protein |
| CT_089 | CopN |
| CT_105 |  |
| CT_115 | Inc protein |
| CT_116 | Inc protein |
| CT_118 | Inc protein |
| CT_119 | Inc protein |
| CT_135 | Inc protein |
| CT_142 |  |
| CT_143 |  |
| CT_156 | lda1 |
| CT_161 |  |
| CT_163 | lda2 |
| CT_166 |  |
| CT_192 | Inc protein |
| CT_195 | Inc protein |
| CT_196 | Inc protein |
| CT_214 | Inc protein |
| CT_222 | Inc protein |
| CT_223 | Inc protein |
| CT_224 | Inc protein |
| CT_226 | Inc protein |
| CT_227 | Inc protein |
| CT_228 | Inc protein |
| CT_229 | Inc protein |
| CT_233 | Inc protein |
| CT_249 | Inc protein |
| CT_257 | lda4 |
| CT_288 | Inc protein |
| CT_300 | Inc protein |
| CT_311 |  |
| CT_324 | Inc protein |
| CT_338 |  |
| CT_345 | Inc protein |
| CT_357 | Inc protein |
| CT_358 | Inc protein |
| CT_365 | Inc protein |
| CT_373 | Inc protein |
| CT_383 | Inc protein |
| CT_429 |  |
| CT_440 | Inc protein |
| CT_441 | Tsp |
| CT_442 | Inc protein |
| CT_449 | Inc protein |
| CT_456 | Tarp |
| CT_473 | lda3 |
| CT_483 | Inc protein |
| CT_529 | Inc protein |
| CT_550 | Inc protein |
| CT_578 | Inc protein |
| CT_579 | Inc protein |
| CT_606.1 | Inc protein |
| CT_610 | Inc protein |
| CT_618 | Inc protein |
| CT_620 |  |
| CT_621 |  |
| CT_622 |  |
| CT_642 | Inc protein |
| CT_652.1 | Inc protein |
| CT_656 |  |
| CT_671 | Inc protein |
| CT_694 |  |
| CT_711 |  |
| CT_712 | Inc protein |
| CT_718 | Inc protein |
| CT_737 | Nue |
| CT_738 | Inc protein |
| CT_789 | Inc protein |
| CT_795 |  |
| CT_798 | GlgA |
| CT_812 | PmpD |
| CT_813 |  |
| CT_823 | CHtrA |
| CT_847 |  |
| CT_848 | Inc protein |
| CT_849 |  |
| CT_850 | Inc protein |
| CT_858 | Cpaf |
| CT_860 | Inc protein |
| CT_861 | Inc protein |
| CT_863 | Inc protein |
| CT_867 | ChlaDub1 |
| CT_868 | ChlaDub2 |
| CT_875 | TepP |

**Table S9:** Ka/Ks ratios of the *pmp* gene family.

| **Gene** | **LocusTag** | **Ka/KS** |
| --- | --- | --- |
| *pmpA* | CT_412 | 0.6409 |
| *pmpB* | CT_413 | 0.679864 |
| *pmpC* | CT_414 | 1.04975 |
| *pmpD* | CT_812 | 0.410611 |
| *pmpE* | CT_869 | 0.26265 |
| *pmpF* | CT_870 | 0.295368 |
| *pmpG* | CT_871 | 0.152529 |
| *pmpH* | CT_872 | 0.124729 |
| *pmpI* | CT_874 | 0.340581 |

**Table S10: NGS library concentration.** Qubit in ng/μL and mean length per sample in bp.

| **Data set** | **NGS library con-**  **centration Qubit in**  **ng/μL** | **Average**  **length in bp** |
| --- | --- | --- |
| CtrE-103 | 1.175 | 883 |
| CtrE-160 | 1.075 | 924 |
| CtrE-32931 | 0.805 | 831 |
| CtrE-547 | 0.746 | 1170 |
| CtrE-8873 | 0.957 | 1051 |
| CtrE-DK-20 | 1.215 | 491 |
| CtrF-6068 | 0.936 | 980 |

**Table S11: *De-novo* assembly results.** The number of contigs and scaffolds together with the N50 for both assemblies. The N50 of MaSuRCA is quite heterogeneous but for five out of the seven samples, Spades assembled nearly the whole genome of 1,04 Mb into one contig. The last two columns show the number of contigs resulting from the comparative assembly and their N50-values.

| **Data set** | **MaSuRCA** | | | **Spades** | | **AMOScmp** | |
| --- | --- | --- | --- | --- | --- | --- | --- |
|  | **Contigs** | **Scaffolds** | **N50** | **Contigs** | **Scaffolds** | **N50** | **Contigs** |
| CtrE-103 | 7 | 7 | 246,067 | 200 | 200 | 1,015,639 | 1 |
| CtrE-160 | 176 | 168 | 30,837 | 1,114 | 1,113 | 53,560 | 1 |
| CtrE-32931 | 29 | 29 | 76,917 | 128 | 128 | 1,021,538 | 2 |
| CtrE-547 | 65 | 63 | 52,095 | 769 | 769 | 1,015,624 | 1 |
| CtrE-8873 | 16 | 16 | 128,930 | 1,294 | 1,294 | 1,042,736 | 1 |
| CtrE-DK-20 | 57 | 49 | 670,938 | 429 | 429 | 138,288 | 3 |
| CtrF-6068 | 37 | 37 | 79,502 | 389 | 389 | 1,015,314 | 1 |

**Table S12: *C. trachomatis* genomes from public databases.** *C. trachomatis* genome sequences used for phylogenetic reconstruction and recombination analysis.

| **GI number** | **NCBI Reference Sequence** | **Description** | **Name** |
| --- | --- | --- | --- |
| 478458543 | NC_020966.1 | *Chlamydia trachomatis* A/363 complete genome | Ctr_A_363 |
| 478448616 | NC_020939.1 | Chlamydia trachomatis A/5291 complete genome | Ctr_A_5291 |
| 478465761 | NC_020944.1 | Chlamydia trachomatis A/7249 complete genome | Ctr_A_7249 |
| 76788711 | NC_007429.1 | Chlamydia trachomatis A/HAR-13, complete genome | Ctr_A_HAR_13 |
| 376282008 | NC_016798.1 | Chlamydia trachomatis A2497, complete genome | Ctr_A2497_1 |
| 385269641 | NC_017437.1 | Chlamydia trachomatis A2497 chromosome, complete genome | Ctr_A2497_2 |
| 237802433 | NC_012686.1 | Chlamydia trachomatis B/Jali20/OT chromosome, complete genome | Ctr_B_Jali20_OT |
| 237804348 | NC_012687.1 | Chlamydia trachomatis B/TZ1A828/OT chromosome, complete genome | Ctr_B_TZ1A828_OT |
| 568111252 | NC_023060.1 | Chlamydia trachomatis C/TW-3, complete genome | Ctr_C_TW_3 |
| 532423942 | NC_022119.1 | Chlamydia trachomatis strain D/13-96, complete genome | Ctr_D_13-96_Seattle |
| 532352287 | NC_022109.1 | Chlamydia trachomatis strain D/14-96 genome | Ctr_D_14-96_Seattle |
| 385243207 | NC_017434.1 | Chlamydia trachomatis D-EC chromosome, complete genome | Ctr_D_EC |
| 385244087 | NC_017436.1 | Chlamydia trachomatis D-LC chromosome, complete genome | Ctr_D_LC |
| 478472060 | NC_020967.1 | Chlamydia trachomatis D/SotonD1 high quality draft genome sequence | Ctr_D_SotonD1 |
| 478454931 | NC_020943.1 | Chlamydia trachomatis D/SotonD5 complete genome | Ctr_D_SotonD5 |
| 478459453 | NC_020968.1 | Chlamydia trachomatis D/SotonD6 high quality draft genome sequence | Ctr_D_SotonD6 |
| 15604717 | NC_000117.1 | Chlamydia trachomatis D/UW-3/CX, complete genome | Ctr_D_UW_3_CX |
| 385241358 | NC_017431.1 | Chlamydia trachomatis E/11023 chromosome, complete genome | Ctr_E_11023 |
| 532351363 | NC_022108.1 | Chlamydia trachomatis strain E/12-94 genome | Ctr_E_12-94_Seattle |
| 385244967 | NC_017439.1 | Chlamydia trachomatis E/150 chromosome, complete genome | Ctr_E_150 |
| 478461249 | NC_020971.1 | Chlamydia trachomatis E/Bour complete genome | Ctr_E_Bour |
| 478472964 | NC_020969.1 | Chlamydia trachomatis E/SotonE4 complte genome | Ctr_E_SotonE4 |
| 478455841 | NC_020942.1 | Chlamydia trachomatis E/SotonE8 high quality draft genome | Ctr_E_SotonE8 |
| 389858571 | NC_017952.1 | Chlamydia trachomatis E/SW3, complete genome | Ctr_E_SW3 |
| 386262357 | NC_017441.1 | Chlamydia trachomatis Sweden2, complete genome | Ctr_E_Sweden2 |
| 532419034 | NC_022117.1 | Chlamydia trachomatis strain F/1-93, complete genome | Ctr_F_1-93_Seattle |
| 532350434 | NC_022107.1 | Chlamydia trachomatis F/11-96, complete genome | Ctr_F_11-96_Seattle |
| 532349507 | NC_022106.1 | Chlamydia trachomatis strain F/2-93 genome | Ctr_F_2-93_Seattle |
| 532421823 | NC_022118.1 | Chlamydia trachomatis strain F/6-94, complete genome | Ctr_F_6-94_Seattle |
| 478473867 | NC_020964.1 | Chlamydia trachomatis F/SotonF3 high quality draft genome sequence | Ctr_F_SotonF3 |
| 389857697 | NC_017951.1 | Chlamydia trachomatis F/SW4, complete genome | Ctr_F_SW4 |
| 389859448 | NC_017953.1 | Chlamydia trachomatis F/SW5, complete genome | Ctr_F_SW5 |
| 385245895 | NC_017440.1 | Chlamydia trachomatis G/11074 chromosome, complete genome | Ctr_G_11074 |
| 385240430 | NC_017430.1 | Chlamydia trachomatis G/11222 chromosome, complete genome | Ctr_G_11222 |
| 385242285 | NC_017432.1 | Chlamydia trachomatis G/9301 chromosome, complete genome | Ctr_G_9301 |
| 385239509 | NC_017429.1 | Chlamydia trachomatis G/9768 chromosome, complete genome | Ctr_G_9768 |
| 478456739 | NC_020941.1 | Chlamydia trachomatis G/SotonG1 complete genome | Ctr_G_SotonG1 |
| 532427121 | NC_022120.1 | Chlamydia trachomatis strain Ia20-97 genome | Ctr_Ia_20-97_Seattle |
| 478460354 | NC_020970.1 | Chlamydia trachomatis Ia/SotonIa1 complete genome | Ctr_Ia_SotonIa1 |
| 478474769 | NC_020940.1 | Chlamydia trachomatis Ia/SotonIa3 complete genome | Ctr_Ia_SotonIa3 |
| 471328175 | NC_020511.1 | Chlamydia trachomatis IU824 complete genome | Ctr_IU824 |
| 478428135 | NC_020512.1 | Chlamydia trachomatis IU888 complete genome | Ctr_IU888 |
| 532353205 | NC_022110.1 | Chlamydia trachomatis strain J/27-97 genome | Ctr_J_27-97_Seattle |
| 532431171 | NC_022121.1 | Chlamydia trachomatis strain J/31-98, complete genome | Ctr_J_31-98_Seattle |
| 527322501 | NC_021892.1 | Chlamydia trachomatis J/6276tet1, complete genome | Ctr_J_6276tet1 |
| 478457650 | NC_020965.1 | Chlamydia trachomatis K/SotonK1 complete genome | Ctr_K_SotonK1 |
| 478451325 | NC_020929.1 | Chlamydia trachomatis L1/115 complete genome | Ctr_L1_115 |
| 478468469 | NC_020972.1 | Chlamydia trachomatis L1/1322/p2 high quality draft genome | Ctr_L1_1322_p2 |
| 478462164 | NC_020973.1 | Chlamydia trachomatis L1/224 complete genome | Ctr_L1_224 |
| 478447723 | NC_020937.1 | Chlamydia trachomatis L1/440/LN complete genome | Ctr_L1_440_LN |
| 478446832 | NC_020930.1 | Chlamydia trachomatis L2/25667R complete genome | Ctr_L2_25667R |
| 166153973 | NC_010287.1 | Chlamydia trachomatis 434/Bu chromosome, complete genome | Ctr_L2_434_Bu |
| 482546028 | NC_021052.1 | Chlamydia trachomatis L2/434/Bu(f), complete genome | Ctr_L2_434_Bu_f |
| 482545086 | NC_021050.1 | Chlamydia trachomatis L2/434/Bu(i), complete genome | Ctr_L2_434_Bu_i |
| 478469369 | NC_020938.1 | Chlamydia trachomatis L2b/795 complete genome | Ctr_L2b_795 |
| 478470269 | NC_020945.1 | Chlamydia trachomatis L2b/8200/07 complete genome | Ctr_L2b_8200_07 |
| 478466660 | NC_020933.1 | Chlamydia trachomatis L2b/Ams1 complete genome | Ctr_L2b_Ams1 |
| 478449525 | NC_020934.1 | Chlamydia trachomatis L2b/Ams2 high quality draft genome sequence | Ctr_L2b_Ams2 |
| 478464861 | NC_020977.1 | Chlamydia trachomatis L2b/Ams3 high quality draft genome sequence | Ctr_L2b_Ams3 |
| 478467560 | NC_020978.1 | Chlamydia trachomatis L2b/Ams4 high quality draft genome sequence | Ctr_L2b_Ams4 |
| 478450425 | NC_020936.1 | Chlamydia trachomatis L2b/Ams5 high quality draft genome sequence | Ctr_L2b_Ams5 |
| 478452224 | NC_020935.1 | Chlamydia trachomatis L2b/Canada1 high quality draft genome sequence | Ctr_L2b_Canada1 |
| 478453138 | NC_020932.1 | Chlamydia trachomatis L2b/Canada2 high quality draft genome sequence | Ctr_L2b_Canada2 |
| 478463961 | NC_020976.1 | Chlamydia trachomatis L2b/CV204 high quality draft genome sequence | Ctr_L2b_CV204 |
| 478471160 | NC_020975.1 | Chlamydia trachomatis L2b/LST high quality draft genome sequence | Ctr_L2B_LST |
| 352951305 | NC_010280.2 | Chlamydia trachomatis L2b/UCH-1/proctitis chromosome, complete genome | Ctr_L2b_UCH_1_proctitis |
| 478454031 | NC_020931.1 | Chlamydia trachomatis L2b/UCH-2 complete genome | Ctr_L2b_UCH_2 |
| 339625373 | NC_015744.1 | Chlamydia trachomatis L2c chromosome, complete genome | Ctr_L2c |
| 478463063 | NC_020974.1 | Chlamydia trachomatis L3/404/LN complete genome | Ctr_L3_404_LN |

**References**

1. Jorgensen, I. and R.H. Valdivia, *Pmp-like proteins Pls1 and Pls2 are secreted into the lumen of the Chlamydia trachomatis inclusion.* Infect Immun, 2008. **76**(9): p. 3940-50.

2. Sisko, J.L., et al., *Multifunctional analysis of Chlamydia-specific genes in a yeast expression system.* Mol Microbiol, 2006. **60**(1): p. 51-66.

3. Lei, L., et al., *Identification of a novel nuclear localization signal sequence in Chlamydia trachomatis-secreted hypothetical protein CT311.* PLoS One, 2013. **8**(5): p. e64529.

4. Vromman, F., et al., *The DUF582 Proteins of Chlamydia trachomatis Bind to Components of the ESCRT Machinery, Which Is Dispensable for Bacterial Growth In vitro.* Front Cell Infect Microbiol, 2016. **6**: p. 123.

5. Olsen, A.W., P. Andersen, and F. Follmann, *Characterization of protective immune responses promoted by human antigen targets in a urogenital Chlamydia trachomatis mouse model.* Vaccine, 2014. **32**(6): p. 685-92.

6. Follmann, F., et al., *Antigenic profiling of a Chlamydia trachomatis gene-expression library.* J Infect Dis, 2008. **197**(6): p. 897-905.

7. Sharma, J., et al., *Profiling of human antibody responses to Chlamydia trachomatis urogenital tract infection using microplates arrayed with 156 chlamydial fusion proteins.* Infect Immun, 2006. **74**(3): p. 1490-9.

8. Budrys, N.M., et al., *Chlamydia trachomatis antigens recognized in women with tubal factor infertility, normal fertility, and acute infection.* Obstet Gynecol, 2012. **119**(5): p. 1009-16.

9. da Cunha, M., et al., *Identification of type III secretion substrates of Chlamydia trachomatis using Yersinia enterocolitica as a heterologous system.* BMC Microbiol, 2014. **14**: p. 40.
